# Critical Evaluation of Branch Polarity and Apical Dominance as Dictators of Colony Astogeny in a Branching Coral

**DOI:** 10.1371/journal.pone.0004095

**Published:** 2009-01-01

**Authors:** Lee Shaish, Baruch Rinkevich

**Affiliations:** Israel Oceanographic and Limnological Research, National Institute of Oceanography, Tel Shikmona, Haifa, Israel; Tel Aviv University, Israel

## Abstract

The high morphological resemblance between branching corals and trees, can lead to comparative studies on pattern formation traits, best exemplified in plants and in some cnidarians. Here, 81 branches of similar size of the hermatypic coral *Stylophora pistillata* were lopped of three different genets, their skeletons marked with alizarin red-S, and divided haphazardly into three morphometric treatment groups: (I) upright position; (II) horizontal position, intact tip; and (III) horizontal position, cut tip. After 1 y of *in-situ* growth, the 45 surviving ramets were brought to the laboratory, their tissues removed and their architectures analyzed by 22 morphological parameters (MPs). We found that within 1 y, isolated branches developed into small coral colonies by growing new branches from all branch termini, in all directions. No architectural dissimilarity was assigned among the three studied genets of treatment I colonies. However, a major architectural disparity between treatment I colonies and colonies of treatments II and III was documented as the development of mirror structures from both sides of treatments II and III settings as compared to tip-borne architectures in treatment I colonies. We did not observe apical dominance since fragments grew equally from all branch sides without documented dominant polarity along branch axis. In treatment II colonies, no MP for new branches originating either from tips or from branch bases differed significantly. In treatment III colonies, growth from the cut tip areas was significantly lower compared to the base, again, suggesting lack of apical dominance in this species. Changes in branch polarity revealed genet associated plasticity, which in one of the studied genets, led to enhanced growth. Different genets exhibited canalization flexibility of growth patterns towards either lateral growth, or branch axis extension (skeletal weight and not porosity was measured). This study revealed that colony astogeny in *S. pistillata* is a regulated process expressed through programmed events and not directly related to simple energy trade-off principles or to environmental conditions, and that branch polarity and apical dominance do not dictate colony astogeny. Therefore, plasticity and astogenic disparities encompass a diversity of genetic (fixed and flexible) induced responses.

## Introduction

In multicellular organisms, the level of integration among bodily components dictates the final functional performance of the entire organism [1]–[5]. This is highlighted in a number of sessile modular organisms like trees [6], [7] and a range of marine invertebrate taxa [8]–[12] sharing similar morphometric traits [4], [13], which produce morphological complexities endowed with sets of ecological advantages when compared to unitary organisms [14]. The whole organism architecture is achieved by amalgamating properties at more than a single level of construction, depicting fixed and flexible morphometric rules, phenotypic plasticity and growth patterns that directly affect life-history traits and fitness [4], [15]–[20].

A further challenging topic is the study of organisms' architectures, made of multiple genetically identical modules, at several levels of organization, which are physiologically and structurally integrated [3], [4], [11], [17]–[19], [21]. Of primary importance are branching structures (in plants and animals alike) that elucidate rules and inherent genetic control for bodily architectures [22]. However, whereas the scientific literature often deals with differences in morphologies in branching types [23], [24], very little attention is given to astogeny rules [18], [19], [25]–[27], including the impacts of positional value through tip dominance and branch polarity.

In various modular organisms, including plants [28] and animals [29], [30; although auxine–like agent is yet to be found] one of the physiological properties is apical dominance. Apical dominance is the sum influence exerted by the shoot apex over lateral bud outgrowth or the stem/branch's tip control over distal parts growth. In many plants, the apical tips produce the growth hormone auxin (determines a tissue property called positional value) that promotes cell division and diffuses downward to inhibit growth of lateral bud, which would otherwise compete with the apical tip for light and nutrients [28]. Experimental manipulation that removed the apical tip and its suppressive hormone, allowed the lower dormant parts to develop, some of them becoming the lead axial growth. Working with the Caribbean gorgonian octocoral *Pseudopterogorgia bipinnata*, Sànchez and Lasker [24] found that clipping off the branch tip results in new growth that exceeds normal rates of branching. Similar systems that do not cause structure formation directly but use tissue positional value through tip dominance, have frequently been recorded in variety of hydrozoans (i.e., [30]), in a way termed as ‘shoot meristem-like organ in animals’ [31].

In branching corals, architectural characteristics can be deduced from traits at three hierarchical levels of organization, the individual polyps [5], [32], the individual branches, and the whole colony entity [18], [19]. While coral forms develop through simple iterated replication of individual polyps, they generate extremely complex and broad structures at the branch [21] and at the colony [18], [19] levels. Despite the relative morphological simplicity of modules at the polyp and the branch levels, branching corals may generate complex architectures at the colony level of organization [4], [24], with extreme modes of phenotypic plasticity [18], [19].

To elucidate further the rules that govern colony development in branching forms, we studied plasticity of colony astogeny and branch to colony trajectories in the Indo-Pacific branching coral *Stylophora pistillata* [18], [19]. A detailed study on dozens of one-year old colonial ramets, generated from isolated single branches of 10 genotypes, revealed a single common astogeny plan for *S. pistillata*, characterized by a continuum of architectural design with several distinct stages. Each stage was marked by its own characteristic morphometric parameters. We presupposed [18] that changing of developmental rules during the trajectory from branch to colony could help the colony to cope better with environmental constraints. A follow-up study [19] evaluated the hypothesis that plasticity can be associated with a degree of structural modularity, where colonial architectures are constructed at different levels of coral-colony organization. The present study seeks to explore the regenerative capability at different branch termini (intact/cut tips, bases), and to examine the relationship between morphometric parameters and colony organization in *S. pistillata*. Special attention is given to whether apical dominance and branch orientation are important in ruling colonial architectures of branching-corals'.

## Results

**Figure 1:**
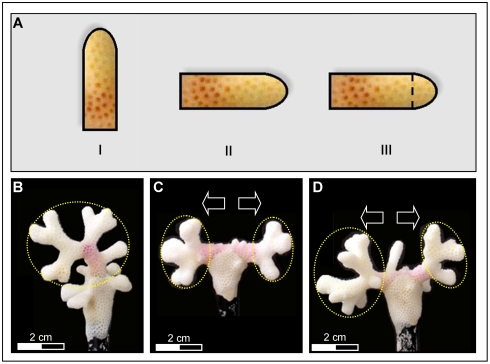
Schematic illustrations describing the initial shape of treatments I to III and representatives of 1 y *in situ* growth. (a) Branch images describing *Stylophora pistillata* treatments types: I, upright position; II, horizontal intact tip; III, horizontal cut tip; (b–d) 1 y *in situ* growth of (b) treatment I, (c) treatment II, and (d) treatment III. Each yellow circle encompasses the volume created by 1 y growth at each branch termini; arrows point to mirror structures developed from both sides of settings II and III.

After 1 y of *in situ* growth, 45 of the 81 fragments (55.6%) survived and developed into colonies of various shapes. These included 15 colonies from treatment I (4, 4, and 7 colonies from genotypes H, I and, J, respectively), 14 colonies from treatment II (4, 4, and 6 colonies from genotypes H, I, and J, respectively) and 16 colonies from treatment III (4, 5 and 7 colonies from genotypes H, I and J, respectively). Upon collection, each colony was dried, measured, and photographed from all angles and 22 morphometric parameters (MPs) were taken (Table 1, average values are given in Table 2). Colonies of all three treatments (Fig. 1a) deposited thin layers of calcium carbonate and tissue material on the plastic tips onto which they were glued (Fig. 1b–d). The material grown on the plastic pin was excluded from analyses.

**Table 1:** Morphometric parameters (MPs) considered as disclosing architectural rules in *S. pistillata* colonial astogeny.

Morphometric character		Description / level of organization	Way of measuring/calculating
**1**	**L 1**	Final length (mm) / Colony	Colony length; measured from one end of the colony to the other.
**2**	**ΔL**	Length added (mm) / Colony	Length added is calculated as: L1-L0.
**3**	**W1**	Final weight of colony (mg) / Colony	Weight after 1 year.
**4**	**ΔW**	Weight added (mg) / Colony	Weight added is calculated as: W1-W0
**5**	**EV**	Ecological volume (mm^3^) / Colony	Sum of skeletal and space between the branches volumes. Calculated as πHr^2^; r = width+length/4. Width and length, see [18].
**6**	**nB**	Total no. of branches / Colony	Total number of branches, including the initial branch [33], [34]
**7**	**TBL**	Total branch length (mm) / Colony	Each branch length was measured by digital caliper to the nearest 0.01 mm. Total length was obtained by summing all lengths of branches [33].
**8**	**Ω**	The order of colony complexity / Colony	According to “Reverse Strahler Order” method [35]; numbers represent the highest order that a specific colony reached.
**9**	**EV/nB**	Branch spacing (mm^3^) / Colony	The ecological volume divided by the total number of branches. Represents the ecological volume per branch.
**10**	**SV**	Skeletal volume / Colony	Sum of all branches' volume (each branch was calculated as a cylinder; according to its length and width measured. [34], [36]
**11**	**SV/EV**	Skeletal to ecological volumes ratio / Colony	Sum of all branches' volume (each branch was calculated as a cylinder) divided by the total ecological volume of the colony.
**12**	**SA**	Surface area of colony / Colony	Summing all branches' surface areas (branch SA was calculated as cylinder surface area.
**13**	**Le**	Lateral extension (mm) / Colony	Colony width, the length between two outer LGB in the colony [18].
**14**	**%N2**	Branches order 2 (%) / Branch	The number of branches from order 2 as part of the total number of branches (Reverse Strahler Order method; [35]).
**15**	**%N3**	Branches order 3 (%) / Branch	The number of branches from order 3 as part of the total number of branches (Reverse Strahler Order method; [35]).
**16**	**%N4**	Branches order 4 (%) / Branch	The number of branches from order 4 as part of the total number of branches (Reverse Strahler Order method; [35]).
**17**	**%UGB**	Up growing branches (%) / Branch	The number of up-growing branches divided by the total number of branches.
**18**	**%LGB**	Lateral growing branches (%) / Branch	The number of lateral branches divided by the total number of branches.
**19**	**%DGB**	Down growing branches (%) / Branch	DGBs were considered as branches growing with their tips facing downwards. The number of down-growing branches is divided by the total number of branches.
**20**	**%SBB**	Stem borne branches / Branch	Number of branches originated from stem only divided by total number of branches in the colony.
**21**	**%TBB**	Tip borne branches / Branch	Number of branches originated from tip only, divided by total number of branches in the colony.
**22**	**%BBB**	Base borne branches / Branch	Number of branches originated from base only, divided by total number of branches in the colony.

**Table 2:** Average (±standard deviation) values of morphometric parameters (MPs) for one-year *in situ* growth of *S. pistillata* colonies. I, II, III - represent treatments types.

		Genet								
		H			I			J		
Preparative		I	II	III	I	II	III	I	II	III
MPs (Colony / Branch level)		Average±SD	Average±SD	Average±SD	Average±SD	Average±SD	Average±SD	Average±SD	Average±SD	Average±SD
Colony	L1 (mm)	44.08±4.02	52.46±5.21	50.81±3.71	39.22±7.06	57.06±3.98	56.93±8.10	41.81±5.56	48.92±6.04	48.63±4.61
	ΔL (mm)	26.79±2.48	27.10±3.11	25.94±3.82	27.95±4.99	34.94±5.10	33.00±7.45	28.68±3.94	24.87±5.24	22.69±3.38
	W1 (gr)	4.69±1.08	6.13±0.53	6.01±1.04	2.96±1.71	7.66±1.76	8.37±1.41	2.94±0.98	4.93±1.41	4.92±0.77
	ΔW (gr)	3.32±1.03	4.09±0.45	3.58±1.16	2.35±1.63	5.86±1.82	6.54±1.11	2.12±0.75	3.04±0.97	2.85±0.62
	EV (mm^3^)	17809±9565	25765±5094	18996±11236	14431±13481	32109±13288	41314±10042	11117±9314	14634±8503	15418±6385
	nB (#)	12.00±4.69	14.25±3.30	13.75±2.99	11.75±5.38	25.25±8.81	28.60±4.10	9.00±4.62	13.00±8.15	10.57±3.55
	TBL (mm)	100.46±29.96	121.61±16.98	111.86±27.69	95.18±49.47	184.11±51.71	204.34±33.59	79.03±30.55	99.99±33.47	91.67±16.18
	Ω	2.50±1.00	3.00±0.00	3.25±0.50	2.50±1.00	4.25±0.96	4.40±0.55	2.57±0.53	3.00±1.10	3.00±0.58
	EV/nB	1482±610	1868±511	1298±562	1059±510	1275±307	1449±322	1089±461	1194±333	1540±572
	SV (mm^3^)	3194±1109	4252±541	3851±1006	2600±2004	6721±1113	7179±1408	2470±775	3850±1314	3516±666
	SV/EV	0.20±0.08	0.17±0.02	0.25±0.12	0.21±0.08	0.24±0.09	0.18±0.02	0.36±0.26	0.31±0.15	0.25±0.05
	SA (mm^2^)	2546±913	3329±501	2979±831	2254±1558	5354±1344	5949±1018	1927±710	2990±1145	2700±609
	Le (mm)	19.40±4.59	22.07±1.56	18.71±6.32	17.40±6.49	23.19±4.65	26.80±2.30	14.92±5.67	16.78±4.69	17.41±2.77
Branch	%N2	0.80±0.19	0.59±0.16	0.51±0.12	0.77±0.23	0.44±0.23	0.37±0.05	0.73±0.08	0.59±0.22	0.68±0.13
	%N3	0.08±0.16	0.34±0.17	0.33±0.10	0.05±0.11	0.32±0.13	0.33±0.08	0.12±0.12	0.23±0.20	0.20±0.14
	%N4	0.00±0.00	0.00±0.00	0.00±0.00	0.08±0.16	0.18±0.18	0.23±0.10	0.00±0.00	0.00±0.00	0.00±0.00
	%UGB	0.21±0.08	0.39±0.14	0.37±0.05	0.24±0.12	0.31±0.08	0.35±0.09	0.44±0.25	0.31±0.07	0.34±0.10
	%LGB	0.78±0.08	0.42±0.14	0.23±0.03	0.72±0.20	0.52±0.11	0.52±0.11	0.56±0.25	0.33±0.20	0.45±0.10
	%DGB	0.01±0.03	0.12±0.06	0.32±0.06	0.04±0.08	0.13±0.03	0.10±0.06	0.00±0.00	0.25±0.15	0.10±0.08
	%SBB	0.79±0.17	0.44±0.18	0.35±0.13	0.75±0.27	0.42±0.30	0.36±0.07	0.66±0.21	0.36±0.19	0.40±0.13
	%TBB	0.21±0.17	0.24±0.09	0.10±0.07	0.25±0.27	0.28±0.09	0.20±0.07	0.34±0.21	0.18±0.12	0.22±0.15
	%BBB	0.00±0.00	0.32±0.11	0.55±0.11	0.00±0.00	0.30±0.25	0.45±0.06	0.00±0.00	0.46±0.23	0.38±0.15

**Figure 2:**
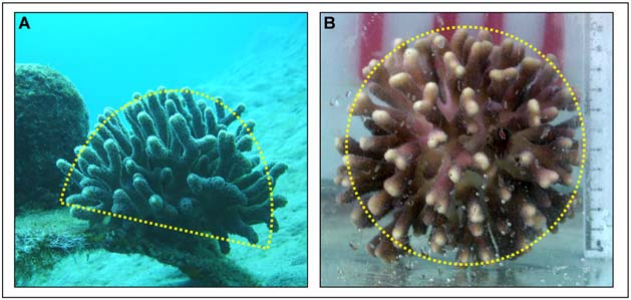
*In situ* developed architectures of *Stylophora pistillata* colonies. (a) The typical hemispherical architecture of a colony growing on natural substrate (photo taken by Y. Horoszowski); (b) A spherical architecture of *S. pistillata* colony growing on the tip of an iron bar. Dash lines depict colony EV.

During this period, treatment I branches grew into small half sphere *S. pistillata* colonies, with UGBs outward and inward LGBs, starting to form the colonial architecture typical of this species (Fig. 2a, 2b). Treatments II and III branches, which were positioned horizontally, developed new branches from both termini (bases, intact tips, and cut-off tips, respectively, Fig. 1c, d). The general architecture of colonies developed from treatments II and III branches, differed from *S. pistillata* colonies grown on natural reef substrates (Fig. 2a) but revealed high similarity to the fully spherical architecture of colonies developed on top of artificial objects (Fig. 2b), including downward trajectories of branches (Fig. 1c, d). Treatment I colonies developed half-sphere structures, most of which were oriented upward and towards the lateral axes (Fig. 1b). Another major architectural disparity between treatment I colonies and colonies of treatments II and III was the growth of mirror structures from both sides of treatments II (Fig. 1c) and III (Fig. 1d) settings (tip and exposed base) compared to tip-borne architectures in treatment I colonies (Fig. 1 b).

**Figure 3:**
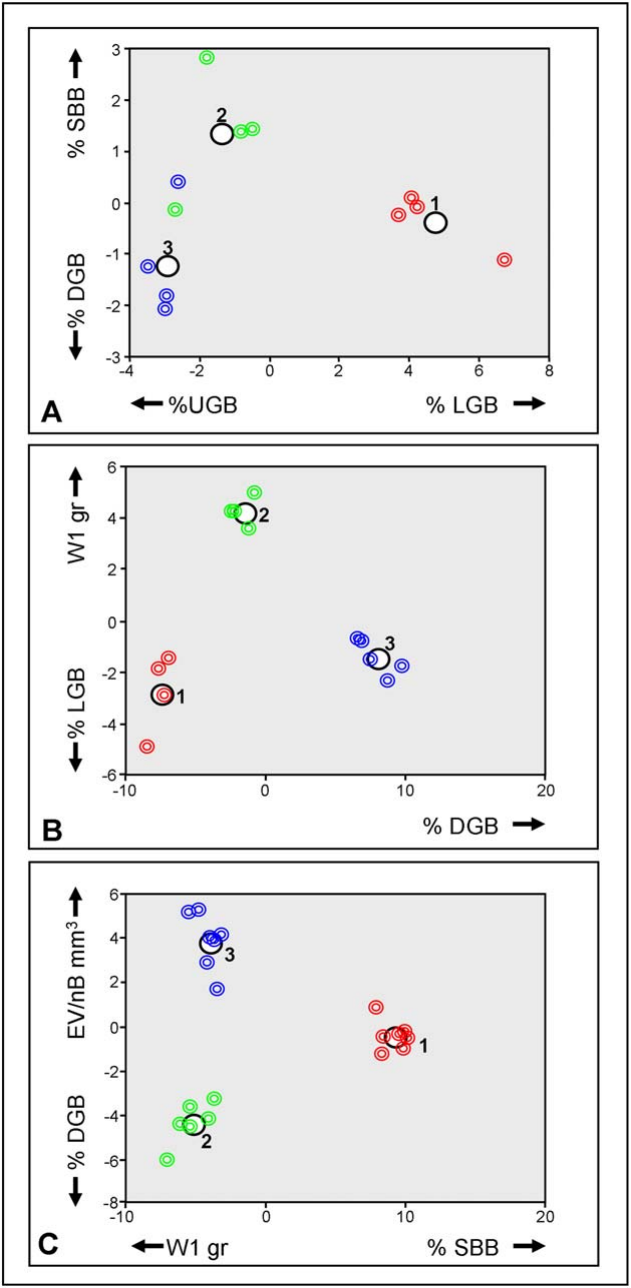
Discriminant analysis results among treatments of each genotype. (a) Genotype H, analysis on the four most discriminating MPs between treatments; (b) Genotype I, analysis on the three most discriminating MPs between treatments; (c) Genotype J, analysis on the four most discriminating MPs between treatments. Red circles are for treatment I, green circles for treatment II, blue circles for treatment III. The larger black circles represent groups centralize.

**Figure 4:**
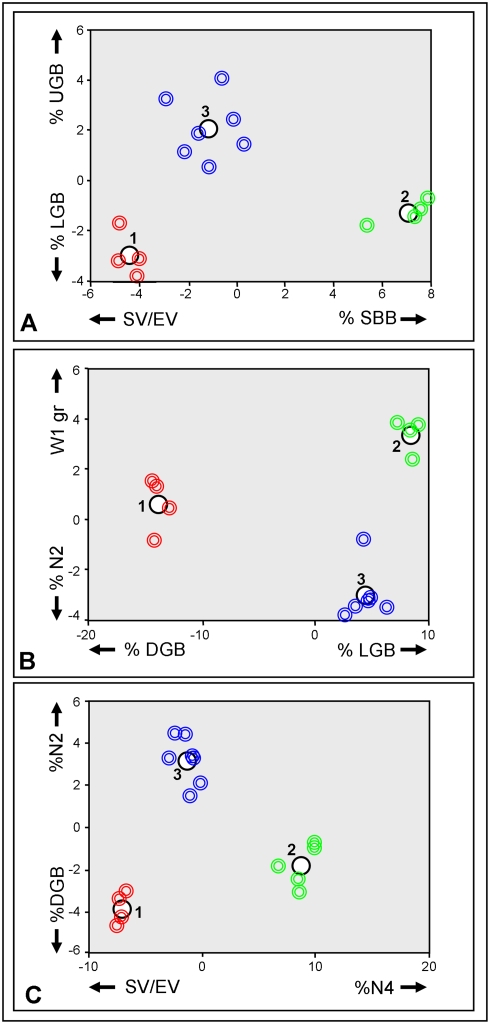
Discriminant analysis results between genotypes for each treatment. (a)Treatment I, the four most discriminating MPs between treatments; (b) Treatment II, the four most discriminating MPs between treatments; (c) Treatment III, the four most discriminating MPs between treatments. Red circles are for genotype H, green circles are for genotype I, blue circles are for genotype J. The larger black circles represent the groups centralize.

First step in the analysis was the conduction of “Pearson Correlation” test, following which MPs: ΔW, TBL, SV, SA and Le, were excluded from the analysis, as resulting with no added information when equated to other MPs. Using ‘Discriminant Analysis test’, the second step in the analysis was the elucidation of MPs that provide highest levels of distinction between the groups. This was preformed first between treatments of each genotype and than between genotypes. Results revealed that in genotype H the most discriminating MPs between treatments are %LGB, %UGB, %DGB and %SBB (Fig. 3a); in genotype I the most discriminating MPs between treatments are %DGB, W1, and %LGB (Fig. 3b); and in genotype J, the MPs W1, %SBB, %DGB and EV/nB (Fig. 3c). Within genotype analyses revealed that for treatment I the most discriminating MPs are %SBB, SV/EV, %UGB and %LGB (Fig. 4a); for treatment II %LGB, %DGB, W1, and %N2 (Fig. 4b); and for treatment III are %N4, SV/EV, %N2 and %DGB (Fig. 4c). The results of the Discriminant Analysis test, therefore, lowered the original selected 22 MPs to nine, as follows: %LGB, %UGB, %DGB, %SBB, %N2, %N4, W1, SV/EV and EV/nB; serving as the most contributing MPs to the discrimination parameters between the groups.

### Differences between treatments in each genotype

**Figure 5:**
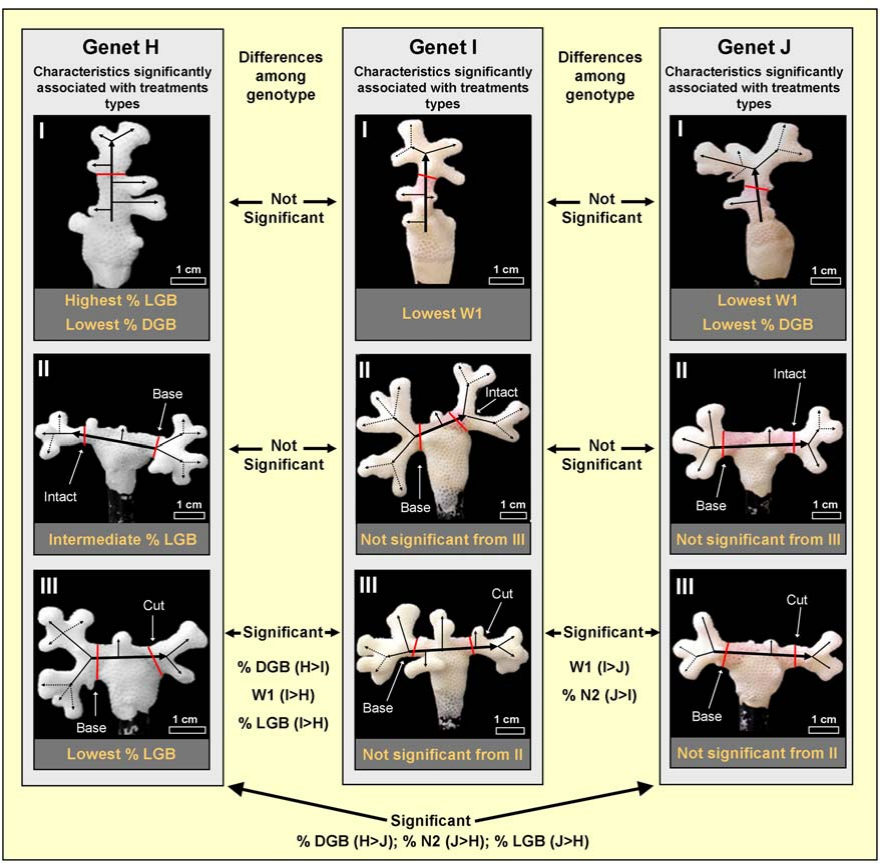
Delineation of the treatment effect within and among genotypes. Growth trajectories in pictures: Thick black lines mark the original branch, arrows reveal initial tip location, thin lines represent N2 branches, dash lines N3. Red lines mark the initial branch size. Intermediate = significantly different from the lowest and the highest values.

Two MPs in genotype H differed between the three treatments. MP %LGB in treatment I colonies (78±8%), treatment II (42±14%) and treatment III (23±3%) differed significantly from each other (ANOVA; p<0.005, LSD; p<0.05, Table 2, Fig. 5). The reverse significant trend was documented for MP %DGB (1±3%, 12±6%, 32±6%, treatments I, II, III, respectively; ANOVA; p<0.005, LSD; p<0.05, Table 2, Fig. 5). In genet I, a significant difference between treatments was found in only one MPs, W1, that was significantly lower in treatment I (2.96±1.71gr) as compared to treatments II (7.66±1.76gr) and III (8.37±1.41gr, ANOVA; p<0.005, LSD; p<0.05, Table 2, Fig. 5), that did not differed significantly (LSD; p>0.05, Table 2, Fig. 5). In genotype J significant differences between treatments were recorded in two MPs. W1 was significantly lower in treatment I (2.94±0.98gr) as compare to treatments II (4.93±1.41gr) and III (4.92±0.77gr, ANOVA; p<0.005, LSD; p<0.05, Table 2, Fig. 5), that did not differed significantly (LSD; p>0.05, Table 2, Fig. 5). Also %DGB was significantly lower in treatment I (0%) as compared to treatments II (25±15%) and III (10±8%, Kruskal-Wallis; p<0.005, Mann-Whitney; p<0.05, Table 2, Fig. 5), that did not differed significantly (Mann-Whitney; p>0.05, Table 2, Fig. 5).

### Differences between genotype in each treatment

For treatments I and II, no significant difference was recorded between the three genotypes, whereas 4 MPs in treatment III significantly differed. W1 was significantly higher in genotype I (8.37±1.41gr) as compare to genotype H (6.01±1.04gr) and J (4.92±0.77gr, ANOVA; p<0.005, LSD; p<0.05, Table 2, Fig. 5), genotypes H and J did not significantly differed (LSD; p>0.05, Table 2, Fig. 5). %N2 was significantly higher in genotype J (68±13%) as compared to genotypes H (51±12%) and I (37±5%, ANOVA; p<0.005, LSD; p<0.05, Table 2, Fig. 5) that did not differed from each other (LSD; p>0.05, Table 2, Fig. 5). %LGB was significantly lower in genotype H (23±3%) as compared to genotypes I (52±11%) and J (45±10%, ANOVA; p<0.005, LSD; p<0.05, Table 2, Fig. 5) that did not differed from each other (LSD; p>0.05, Table 2, Fig. 5). %DGB was significantly higher in genotype H (32±6%) as compared to genotypes I (10±6%) and J (10±8%, ANOVA; p<0.005, LSD; p<0.05, Table 2, Fig. 5) that did not differed from each other (LSD; p>0.05, Table 2, Fig. 5).

### Between/within branch termini- treatments II and III analyses

Treatments II and III were further analyzed for differences in developmental architectures exhibited by the branch termini. Comparison was done in two ways: (1) between tips or bases of the two polarity manipulated treatments (II compared to III); and (2) between tip (either intact or cut-off) and base of the same branch. Five most relevant MPs for this comparisons were chosen: (1) nB (total number of branches developed from tip or from base of the total number of branches in the colony); (2) %nB (percentage of branches developed from tip (TBB) or from base (BBB) of the total number of branches in the colony); (3) TBL (total length of branches developed at either tip or base in a single branch); (4) EV (the ecological volume of developed tip or base, including branches and the spaces between them); and (5) %Nx, the number of branches from orders 2 to 4 as part of the total number of branches (Table 3).

**Table 3:** Average morphometric parameters (MPs) values for developing *S. pistillata* colonies, following 1 y of *in situ* growth. II, III - represent the treatments types; SD = standard deviation.

	Genet					
	H		I		J	
	II	III	II	III	II	III
MPs	Average±SD	Average±SD	Average±SD	Average±SD	Average±SD	Average±SD
#TBB	3.50±1.91	1.50±1.00	6.75±2.63	5.40±1.52	2.33±1.63	2.71±2.21
#BBB	4.75±2.22	7.75±3.10	8.50±7.23	12.80±2.77	6.50±6.89	3.71±1.60
TBL/T	22.33±11.61	12.14±9.16	44.54±17.53	34.69±1.61	14.22±11.40	14.84±10.00
TBL/B	29.30±17.50	45.17±26.13	55.39±46.82	81.40±19.24	33.09±32.51	23.79±9.38
%TBB	0.24±0.09	**0.10±0.07**	0.28±0.09	0.20±0.07	0.18±0.12	0.22±0.15
%BBB	0.32±0.11	**0.55±0.11**	0.30±0.25	0.45±0.06	0.46±0.23	0.38±0.15
EV/T	407±253	416±447	797±498	547±268	**255±277**	**191±127**
EV/B	285±189	696±510	431±358	538±166	**248±131**	**359±281**
%N2t	0.15±0.04	0.10±0.07	0.09±0.043	0.07±0.01	0.13±0.08	0.15±0.07
%N2b	0.15±0.04	0.15±0.04	0.05±0.035	0.07±0.01	0.21±0.15	0.24±0.13
%N3t	0.09±0.11	0.00±0.00	0.14±0.035	0.12±0.07	0.05±0.08	0.07±0.13
%N3b	0.17±0.14	0.31±0.07	0.11±0.083	0.15±0.01	0.17±0.19	0.13±0.13
%N4t	0.00±0.00	0.00±0.00	0.05±0.058	0.01±0.02	0.00±0.00	0.00±0.00
%N4b	0.00±0.00	0.09±0.18	0.11±0.157	0.20±0.08	0.04±0.10	0.01±0.03

Bold numbers depict significant values (p<0.05) of comparisons within the same treatment of a specific genotype.

Comparisons between the treatments revealed no significant differences in any tested MPs between treatments II and III for all the genets.

Comparison within treatment II (between intact tip and exposed base) revealed no significant differences between all five MPs studied in genets H and I (Paired T-test; p>0.006, Table 3). In genet J a significant difference was recorded in the ecological volume created at each end of the branch (EV/T vs. EV/B), which was significantly higher at the tip (255±277 mm^3^) compared to the base (248±131 mm^3^, Paired T-test; p<0.006, Table 3). Comparisons within treatment III (between cut tip and cut base; Table 3) revealed significant differences as follows: within one MPs in genet H (%TBB vs. %BBB; Paired T-test; p<0.006), within non of MPs in genet I (Paired T-test; p>0.05) and one in genet J (EV/T vs. EV/B, Paired T-test; p<0.006, Table 3).

## Discussion

In this study, we strived to find the importance of branch tips (polarity) to coral colony astogeny and to address the possible existence of apical dominance, a well-documented phenomenon in the plant world, in shaping coral architectures. Results of this, and earlier studies on the branching coral *Stylophora pistillata* [4], [18], [19], [37], [38] have depicted fixed and flexible morphometric rules, phenotypic plasticity and growth patterns that directly affected traits associated with coral morphology. While previous studies focused on non-heritable phenotypic variations as protective tools against inclement environmental conditions [6], [7], [23], [35], [39], or as tools protecting the interacting genotypes from selection pressures [40], [20], results of studies on *Stylophora pistillata* astogeny ([4], [18], [19]; this study) revealed the importance of fixed and flexible traits, probably controlled by genetic elements, in shaping colonial architectures.

This study is based on 45 one-year old *Stylophora pistillata* ramets, grown from similar size branches, and sub-cloned from three different coral genets. Ramets were haphazardly divided into three morphometric settings (Fig. 1a), and used in analyses for branch polarity and apical dominance as determining colony astogeny. No known fitness trade-offs associated with coral colony plasticity were imposed on the growing ramets that resided *in situ* for 1 y one next to others on the same substrate and, seemingly, influenced by the same micro-environmental conditions.

Results revealed that while no architectural dissimilarities were assigned to among the three studied genets of treatment I colonies (vertically grown ramets) or treatment II colonies (horizontal grown ramets intact-tip), genotype-based differences emerged in treatments III (horizontal grown ramets with cut-tip). We found that altering branch orientation, in addition of trimming the branch tip (upper 0.5 cm), triggered species-specific and colony-specific architectural reactions. Genotype H exhibited an increase in percentage of down-facing branches (%DGB) as compared to the other two genotypes, genotype I gained more weight (W1) as compared to the other genotypes and genotype J showed an increase in percentage of branch order 2 (%N2) as compared to the other two genotypes. Other MPs, where differences were not yet resulted in significant values (because of high variation in the results) and were noticeable to the eye, were the developed total number of branches (nB) and ecological volume (EV). Genotype H showed 19% and 15% increase in total number of branches when comparing treatment I to II and I to III, respectively, and 45% and 7% in ecological volume, respectively. Genotype I exhibited increases of 115% and 143% in total number of branches when comparing treatment I to II and I to III, respectively, and 123% and 186% in ecological volume, respectively. Genotype J displayed 44% and 17% increase in nB, respectively, 27 and 32% and 39% in EV, respectively. As stated, these values however, were not significantly different from each other's.

While the traditional test for apical dominance in plants entails removal of the apical bud and measuring the effects on the dormant buds lower on the branch [28], [41], the *S. pistillata* mode of astogeny [4], [18], [19] precludes an analogous test (removal of the tip for the appearance of new side branches). Therefore, we use the term ‘apical dominance’ in its broad sense, following studies on gorgonians and hard corals that showed the importance of the apical side of the coral branch in colony astogeny [24], [42]–[47]. Therefore, turning the branch on its side, as done in this study, provides an important add-on to the analysis, as it ‘frees’ the base for potential growth. However, this assay may also reflect impacts of branch orientation. A major architectural disparity between treatment I colonies (naturally posing growth trajectories) and colonies of treatments II and III (horizontally posing growth trajectories) was the development of mirror structures on both sides of treatments II and III settings, compared to the species specific tip-borne architectures developed in treatment I colonies (Fig. 5). Additionally, no apical dominance was recorded, as fragments in treatments II and III grew equally on both branch sides, and no dominant polarity along branch axis was recorded. All MPs studied for new branches developed from branch tips or from branch bases in treatment II colonies, did not differ significantly. The resemblance between the way branch tips and branch bases developed, negated the possibility of apical dominance in *S. pistillata* branching system, as in the case of the soft coral *Nephthea* sp., where cutting the terminal polyps in young colonies did not change the way the colony developed [43]. In treatment III colonies, the rate of growth from the cut tip area was significantly lower compared to the base, further suggesting lack of apical dominance in this species.

The impacts of branch orientation and polarity on morphometric parameters regulating colonial astogeny was revealed earlier by Kawaguti [42] who cited the orientation of branch setting (shaped horizontally or inverted compared to naturally growing branches) as an important factor. As with the *Stylophora* settings II and III in the present study, *Acropora* branches developed terminal polyps at both sides of the branch. However, while in the *Acropora* treatment [42] new polyps tended to grow towards a light source (phototropism), the *Stylophora* treatment did not show any phototropism impact as new branches initiated up and down trajectories, without obvious preference for light.

Changing branch orientation affected the way morphology developed, the complexity of the branching system (represented by numbers of branch generations), and the growth of new branches from branch tip or along the branch. When the initial branch orientation changed from horizontal to vertical, the percentage of high order of branches (N2 compare to N3 or N4) was higher than that of the horizontal branch. Altering the orientation of the branch from vertical to horizontal might result in shifting energy (evident by developing of new branches) from the original branch tip area (intact or cut-tip branch) to the opposite branch end, the base. It is of major interest to note that new branches developed from both tip ends rather than from the whole length of the branch, resembling induced positional information [1]. In contrast, during the *Stylophora* species-specific colonial astogeny from vertical oriented branches, most new branch initiations developed along the branch.

Astogeny of cut branch in *Stylophora pistillata* is highly regulated as in whole colony scenarios [37], [44]–[48]. Cutting off branch tips led to enhanced new growth from branch bases compared to horizontally positioned branches with intact tips. This contrasts results from other branching species like *Acropora millepora* and *Pocillopora damicornis* [49], but brings to mind the responses recorded in other cnidarians. Six months after cutting the tip of the main branch from a colonial gorgonian [24], the lower part of the colony started to grow many new side-branches, from which new lateral branches developed and new axes of growth appeared, indicating the existence of ‘dormant points of initiation’ [24]. We assumed that these dormant points are activated when a major point of initiation (like branch tip) is damaged.

It is evident that coral colonies, isolated branches, and spat not only ‘sense’ environmental cues but also ‘discern’ their special position. Working on two branching coral species, including *Stylophora pistillata*, Meroz et al. [50], documented that coral orientation may influence pattern formation. They found that spat and adult colonies sense gravity (as do mammalian cells; [51]) that influences morphometric parameters of colonial architectures. By experimentally altering direction and intensity of gravitational forces acting along or perpendicular to the main body axis of the coral polyps, they found that vertically growing polyps had significantly higher slenderness ratios than horizontal settings. As revealed by the present results on branch settings, other morphometric parameters in the study by Meroz et al. [50], such as polyp volume, dry skeleton weight, and density, were not flexible and did not vary significantly under altered gravity direction and intensity. Therefore, even an omnipresent force like gravity, may depict deviations between fixed vs. flexible morphometric parameters. Regeneration is a regulated process expressed through programmed events and not directly related to the energy trade-off principle. Therefore, plasticity and astogenic disparities encompass a diversity of fixed and flexible induced responses.

## Materials and Methods

### Species studied

The Indo-Pacific branching coral *Stylophora pistillata* (Esper, 1787; Fig. 2a, b) is a fast growing and important reef builder, characterized by sphere-like architectural symmetry and a variety of color morphs [4]. Polyps (each approximates 1 mm in diameter) are added by inter-tentacular budding. Astogeny is arranged by axial growth form of existing branches and by integrated developmental processes of new up-growing branches (UGBs) that are added, primarily, by dichotomous fission at a branch-tip and inward- and outward-facing lateral branches (LGBs), together forming the three-dimensional (3D) half sphere (Fig. 2a) or spherical symmetry (Fig. 2b). The apex of each UGB or LB axis comprises several contiguous polyps. While inward-facing LGBs cease to grow at a certain point, avoiding isogenic fusions with UGB branches, outward-facing LGBs develop similarly to UGBs, adding more ecological volume to the colony's spherical structure [4], [48], [52]. As in other coral species [5], the developing *Stylophora pistillata* colony responds to the environment by sets of morphometric rules [18], [19] that “canalize” (sensu [53], [54] growth patterns to the typical species morphology.

### Experiment conducted

*In situ* experiments on ramets taken from three large *Stylophora pistillata* colonies were conducted in front of the H. Steinitz Marine Biology Laboratory at Eilat, the northern Red Sea, at a depth of 7 m. and at 10 m distance from each other. The colonies (15–20 cm diameter each; marked as H, I, J), were carefully detached from the substrates by chisel and hammer. Each colony represented only a single genet, as colonial fragments of this species in Eilat do not resume development [19]. Following collection, colonies were incubated *in situ* in clear plastic bags with alizarin Red S solution (15 ppm; 12 h; following Rinkevich, [48]). Tips of branches, the major site of calcification, were marked during labeling by red color, whereas newly deposited calcium carbonate areas appeared as white zones above the red lines. After two weeks of post-labeling acclimation period, 27 single-tip branches (ramets), 2–4 cm long apiece, were removed from each colony by wire cutter. Ramets from each *Stylophora* genotype were divided into three settings/treatments (Fig. 2a): (I) single tip branches fixed to small plastic pins in upright position, (II) intact single tip branches attached to small plastic pins in horizontal position (7 cm above substrate), and (III) half-centimeter cut off single tip branches, attached to small plastic pins in horizontal position (7 cm above substrate). All branches were affixed to a plastic pin by Aqua-Mend underwater epoxy glue, the plastic pins were held by clips to underwater nursery tables, placed at 7 m depth, 1 m above substrate under identical in situ conditions. The surviving ramets that developed into small colonies were brought to the laboratory and their tissues removed by immersion in household bleach for 24 h [46].

Twenty-two morphometric parameters (MPs; Table 1) were measured and analyzed for each colony. The MPs were divided into two groups; (1) those describing the characteristics at the colony level (L1, ΔL, W1, ΔW, EV, nB, TBL, Ω, SV, SV/EV, SA and Le), and (2) those describing properties of branching architecture (%N2, %N3, %N4, EV/nB, %UGB, %LGB, %DGB, %SBB, %TBB and %BBB). Not all morphometric parameters were analyzed for each setting, as some were deemed redundant or irrelevant.

### Data analysis

First step in the analysis carried out “Pearson Correlation”, in order to remove parameters that are related (p>0.9), therefore do not add information to the analysis. The second step of the analysis was to elucidate those MPs that provide the best discrimination between the groups, using Discriminant Analysis test. This analysis was preformed initially within each genotype between treatments and than, on each treatment between genotypes and for each test, three to four most discriminating MPs were chosen. Following that, an ANOVA was performed on the selected MPs checking for significance of differences, first in each genotype between treatments and than, in each treatment between genotypes. The level of significant was calculated using Bonferoni correction to avoid type I error. In the ANOVA tests, the preliminary assumption was the existence of homogeneity of variance and not normal distribution [55]. Homogeneity of variance was checked using Levene's test. When no homogeneity of variance was found, transformation of square root or Log 10 was preformed on the data [56]. The a-parametric tests Kruskal-Wallis and Mann-Whitney were used for cases when transformation did not reveal homogeneity of variance. Tip vs. base comparisons of the same colony were performed by a Paired Samples Test. We performed Wilcoxon Signed Rank Test for situations of non-homogeneity of variance.
